# Supplementary material for: Development of DHODH inhibitors incorporating virtual screening, pharmacophore modeling, fragment-based optimization methods, ADMET, molecular docking, molecular dynamics, PCA analysis, and free energy landscape
Source: PLoS One. 2026 Feb 23;21(2):e0342461. doi: 10.1371/journal.pone.0342461 (PMC12928574; doi:10.1371/journal.pone.0342461)
Supplement: S1 Table — (DOCX) [file pone.0342461.s001.docx]

**Table S1.** Small molecules that form pharmacophore models

| **Molecule** | **Smiles** |
| --- | --- |
| 1 | BrC1CCC(C2CSC(NNCC3CCCCC3)N2)CC1 |
| 5 | OC(O)C1CCCCC1NNCC1CCC(C2CCCCC2Cl)O1 |
| 18 | COC1CCCC(C(O)NC2NC3CCCCC3S2)C1 |
| 20 | NCC(C(O)NC1CCC(C2CCCCC2)CC1)C(O)C1CC1 |
| 21 | OC1C2CCCCC2C(O)C([C@H]2CC[C@@H](C3CCC(Cl)CC3)CC2)C1O |
| 24 | CN(C)C1CCC(C2CCC(NC(O)C3CCCC3C(O)O)CC2Cl)CC1 |
| 25 | OC(O)C1CCCC1C(O)NC1CCC(C2CCC(OC(F)(F)F)CC2)CC1F |
| 26 | NCC1CC(C2CCCC(OC(F)(F)F)C2)CCC1NC(O)C1CCCC1C(O)O |
| 27 | COC1CCCC(C2CCC(NC(O)C3CCCC3C(O)O)C(OC)C2)C1 |
| 28 | OC(O)C1CCCC1C(O)NC1CCC(C2CCCC(OC(F)(F)F)C2)CC1F |
| 30 | CN(C)C1CCC(C2CC(F)C(NC(O)C3CCCC3C(O)O)C(F)C2)CC1 |
| 32 | OC(O)C1CCCC1C(O)NC1CCC(C2CCC(Br)CC2)CC1Cl |
| 33 | [O-][NH+](O)C1CC(C2CCCC(OC(F)(F)F)C2)CCC1NC(O)C1CCCC1C(O)O |
| 36 | COC1CC(C2CCCCC2Cl)CCC1NC(O)C1CCCC1C(O)O |
| 37 | OC(O)C1CCCC1C(O)NC1CCC(C2CCCC(OC(F)(F)F)C2)CC1C(F)(F)F |
| 47 | CCC(O)[C@H](CN)C(O)NC1CCC(C2CCCCC2)C(C(O)OC)C1 |
| 48 | CCC(O)C(CN)C(O)NC1CCC(C2CCC(F)CC2)C(C(O)OC)C1 |
| 49 | CCC(O)C(CN)C(O)NC1CCC(C2CCCCC2Cl)C(C(O)OC)C1 |
| 50 | CCC(O)C(CN)C(O)NC1CCC(C2CCCC(Cl)C2)C(C(O)OC)C1 |
| 51 | CCC(O)C(CN)C(O)NC1CCC(C2CCC(Cl)CC2)C(C(O)OC)C1 |
| 52 | CCC(O)C(CN)C(O)NC1CCC(C2CCCC(OC(F)(F)F)C2)C(C(O)OC)C1 |
| 53 | CCC(O)C(CN)C(O)NC1CCC(C2CCCC(F)C2)C(C(O)OC)C1 |
| 54 | CCC(O)C(CN)C(O)NC1CCC(C2CCC(F)CC2F)C(C(O)OC)C1 |
| 56 | CCC(O)C(CN)C(O)N[C@H]1CC[C@@H](C2CCC(F)CC2C)C(C(O)OC)C1 |
| 57 | CCC(O)C(CN)C(O)NC1CCC(C2CCC(F)NC2)C(C(O)OC)C1 |
| 59 | CCC(O)C(CN)C(O)NC1CCC(C2CC(F)C(F)C(F)C2)C(C(O)OC)C1 |
| 60 | CCC(O)C(CN)C(O)NC1CCC(C2CCC(F)CC2OC)C(C(O)OC)C1 |
| 63 | CCC(O)C(CN)C(O)NC1CC[C@@H](C2CCCC(OC)C2)C(C(O)OC)C1 |
| 64 | CCC(O)C(CN)C(O)NC1CCC(C2CCC3OCCOC3C2)C(C(O)OC)C1 |
| 65 | CCC(O)C(CN)C(O)NC1CCC(C2CCCC(OC(F)F)C2)C(C(O)OC)C1 |
