# Supplementary material for: Development of DHODH inhibitors incorporating virtual screening, pharmacophore modeling, fragment-based optimization methods, ADMET, molecular docking, molecular dynamics, PCA analysis, and free energy landscape
Source: PLoS One. 2026 Feb 23;21(2):e0342461. doi: 10.1371/journal.pone.0342461 (PMC12928574; doi:10.1371/journal.pone.0342461)
Supplement: S2 Table — (DOCX) [file pone.0342461.s002.docx]

**Table S2.** Active molecule

| **Molecule** | **Smiles** |
| --- | --- |
| 31 | OC(O)C1CCCC1C(O)NC1C(F)C(F)C(C2CCCC(OC(F)(F)F)C2)C(F)C1F |
| 34 | OC(O)C1CCCC1C(O)NC1CCC(C2CCCC(OC(F)(F)F)C2)CC1Cl |
| 39 | CC(C)(C)C1CCC(C2CCC(NC(O)C3CCCC3C(O)O)CC2Cl)CC1 |
| 66 | CCC(O)C(CN)C(O)NC1CCC(C2CCCC(OC3CCC3)C2)C(C(O)OC)C1 |
| 69 | COC1CCCC(C2CCC(NC(O)C3CSCC3C(O)O)C(F)C2)C1 |
| 70 | OC(O)C1CSCC1C(O)NC1C(F)CC(C2CCCC(OC(F)(F)F)C2)CC1F |
| 72 | OC(O)C1CSCC1C(O)NC1CC(Br)C(OCC2C(F)CCCC2Cl)C(Br)C1 |
| 73 | OC(O)C1COCC1C(O)NC1C(F)C(F)C(C2CCCC(OC(F)(F)F)C2)C(F)C1F |
| 74 | OC(O)C1CSCC1C(O)NC1C(F)C(F)C(C2CCCC(OC(F)(F)F)C2)C(F)C1F |
| 75 | OC(O)C1COCC1C(O)NC1C(F)CC(C2CCCC(OC(F)(F)F)C2)CC1F |
| 76 | OC(O)C1CSCC1C(O)NC1CC(Br)C(OCC2CCCCC2)C(Br)C1 |
| 77 | COC1CCCC(C2CC(F)C(NC(O)C3CCCC3C(O)O)C(F)C2)C1 |
| 84 | OC(O)C1CS(O)(O)CC1C(O)NC1CCC(C2CCCCC2)CC1 |
| 85 | OC(O)C1CCCC1C(O)NC1CC(Br)C(OCC2C(F)CCCC2Cl)C(Br)C1 |
| 86 | COC1CCCC(C2CCC(NC(O)C3C(C(O)O)CC[C@@H]3O)C(F)C2)C1 |
| 87 | COC1CCCC(C2CCC(NC(O)C3CCCC3C(O)O)C(F)C2)C1 |
| 88 | COC1CCCC(C2CCC(NC(O)C3COCC3C(O)O)C(F)C2)C1 |
| 89 | OC(O)C1CCCC1C(O)NC1CC(Br)C(OCC2CCCCC2)C(Br)C1 |
| 95 | CCOC1CCCC(C2CC(F)C(NC(O)C3CCSC3C(O)O)C(F)C2)C1 |
| 98 | CCOC1CCCC(C2CC(F)C(NC(O)C3COCC3C(O)O)C(F)C2)C1 |
| 159 | CCOC1CCCC(C2CCC(NC3NCCCC3C(O)O)C(F)C2)C1 |
| 160 | COC1CCCC(C2CC(F)C(NC3NCCCC3C(O)O)CC2F)C1 |
| 161 | COC1CCCC(C2CCC(NC3NCCCC3C(O)O)C(Cl)C2)C1 |
| 268 | COC1CCC(C2CCC(NC(O)C3CCCC3C(O)O)C(F)C2)CC1 |
| 269 | OC(O)C1CCCC1C(O)NC1CCC(C2CCCCC2)CC1 |
| 270 | OC(O)C1CCCC1C(O)NC1CCC(C2CCC(Br)CC2)C(Cl)C1 |
| 345 | OC(O)C1CC(C2CC2)CCC1NC1CNC(C2CCCCC2F)NC1 |
| 346 | OC(O)C1CC(C2CC2)CCC1NC1CNC(C2CCCCC2C(F)(F)F)NC1 |
| 347 | CC1CCCCC1C1NCC(NC2CCC(C3CC3)CC2C(O)O)CN1 |
| 348 | OC(O)C1CC(C2CC2)CCC1NC1CNC(C2CC(F)CCC2F)NC1 |
| 349 | OC(O)C1CC(C2CC2)CCC1NC1CNC(C2CC(C(F)(F)F)CCC2F)NC1 |
| 350 | OC(O)C1CC(C2CC2)CCC1NC1CCC(C2CCCCC2F)NC1 |
| 460 | OC(NC1CC1)C1CCC(N2CCSC3CCCCC32)S1 |
| 461 | OC(O)C1CCCC1C(O)NC1CCC(OCC2CCCCC2)CC1 |
| 462 | COC1CCCC(C2CCC(NC(O)C3COCC3C(O)O)C(F)C2)C1 |
| 464 | COC1CCCC(C2CCC(NC(O)C3CCSC3C(O)O)C(F)C2)C1 |
| 465 | OC(O)C1CSCC1C(O)NC1CCC(C2CCCCC2)CC1 |
| 471 | OC(O)C1SCCC1C(O)NC1CCC(C2CCCCC2)CC1 |
