# Supplementary figures and images for: Development of DHODH inhibitors incorporating virtual screening, pharmacophore modeling, fragment-based optimization methods, ADMET, molecular docking, molecular dynamics, PCA analysis, and free energy landscape
Source: PLoS One. 2026 Feb 23;21(2):e0342461. doi: 10.1371/journal.pone.0342461 (PMC12928574; doi:10.1371/journal.pone.0342461)

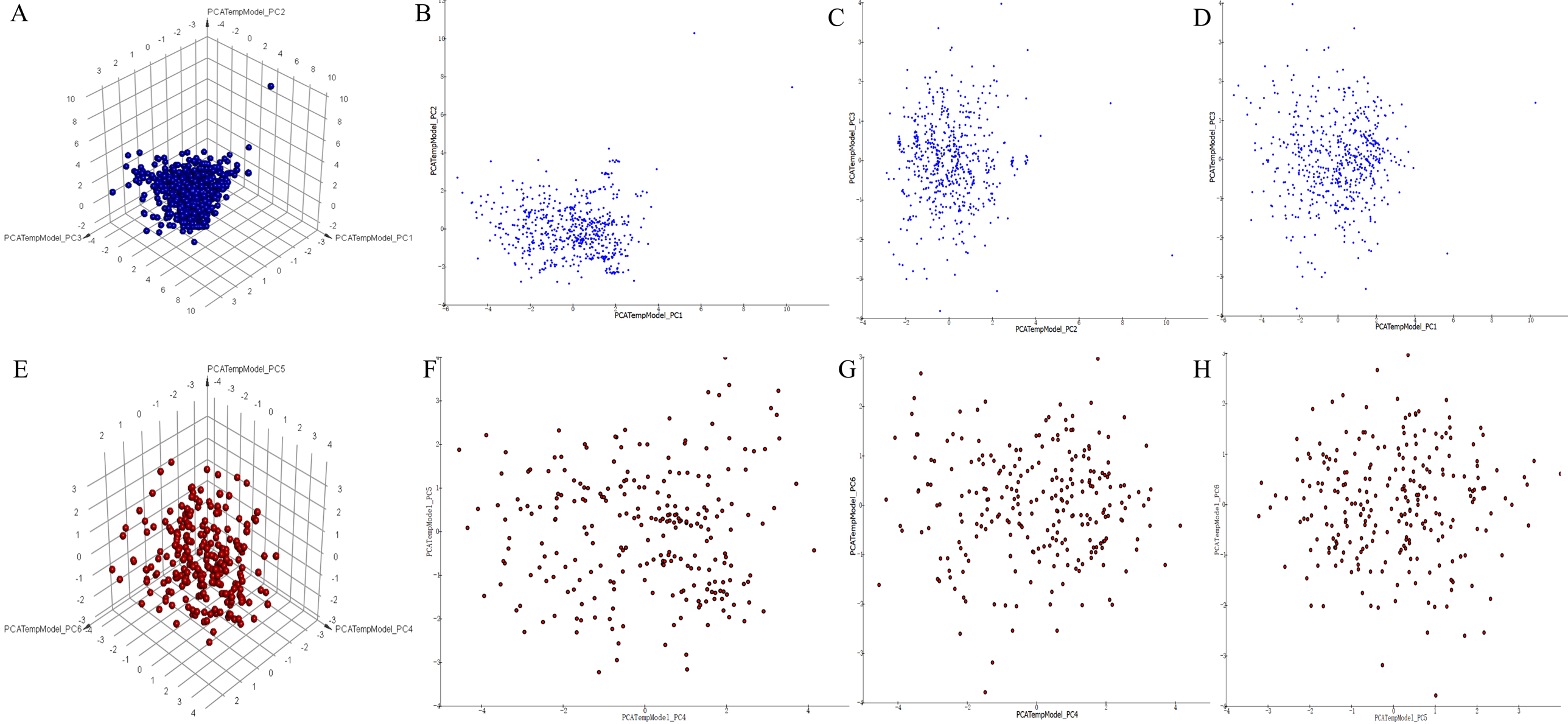

Supplement: S1 Fig — (A, E) are the spatial distribution diagrams of the main components (PC 1-PC 6) of the compound: (B). Two-dimensional spatial distribution of PC1 and PC2, (C). Two-dimensional spatial distribution of PC1 and PC3, (D). Two-dimensional spatial distribution of PC2 and PC3, (F). Two-dimensional spatial distribution of PC4 and PC5, (G). Two-dimensional spatial distribution of PC4 and PC6, (H). Two-dimensional spatial distribution of PC5 and PC6. (TIF) [file pone.0342461.s003.tif]

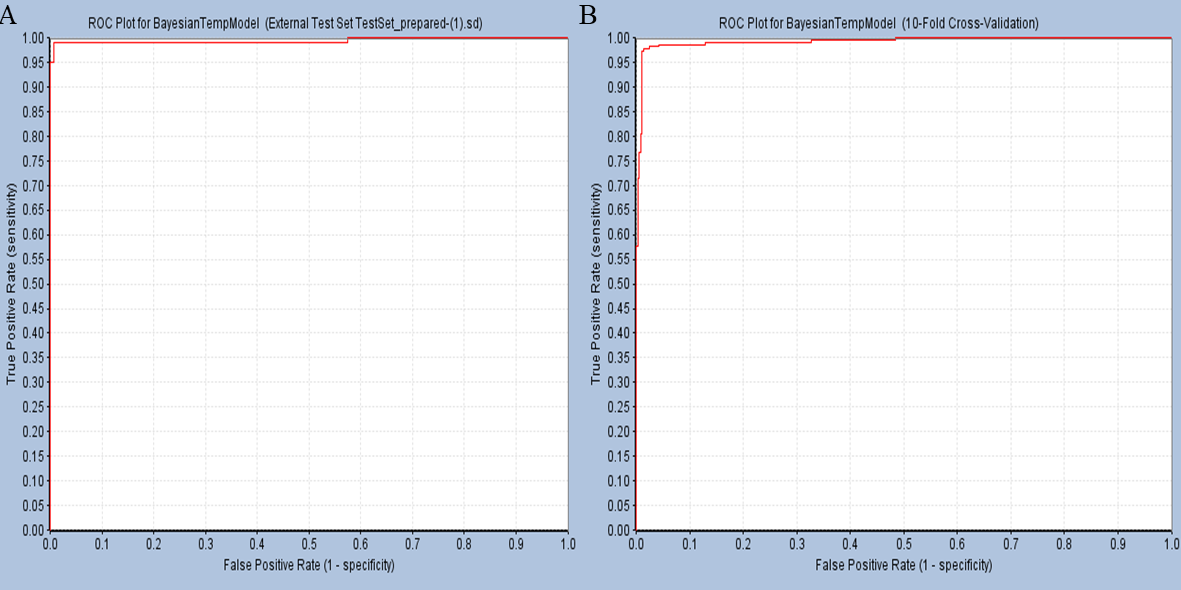

Supplement: S2 Fig — (A): Ten-fold ROC curve of the Bayesian model (B): Multi-set validation ROC curves of the NB model. (TIF) [file pone.0342461.s004.tif]

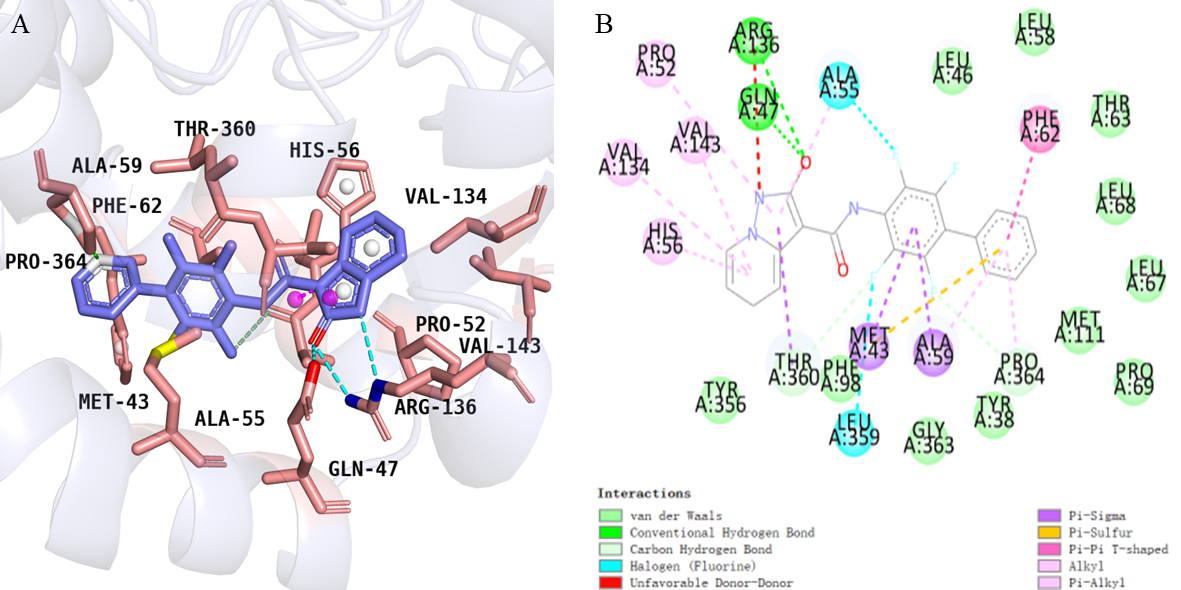

Supplement: S3 Fig — (A) 3D interaction diagram of compoundDUH. Hydrogen bonds are shown as green dashed lines, Hydrophobicity is in red lines. (B) 2D interaction diagram of compound DUH. Hydrogen bonds are shown as purple dashed lines. (TIF) [file pone.0342461.s005.tif]

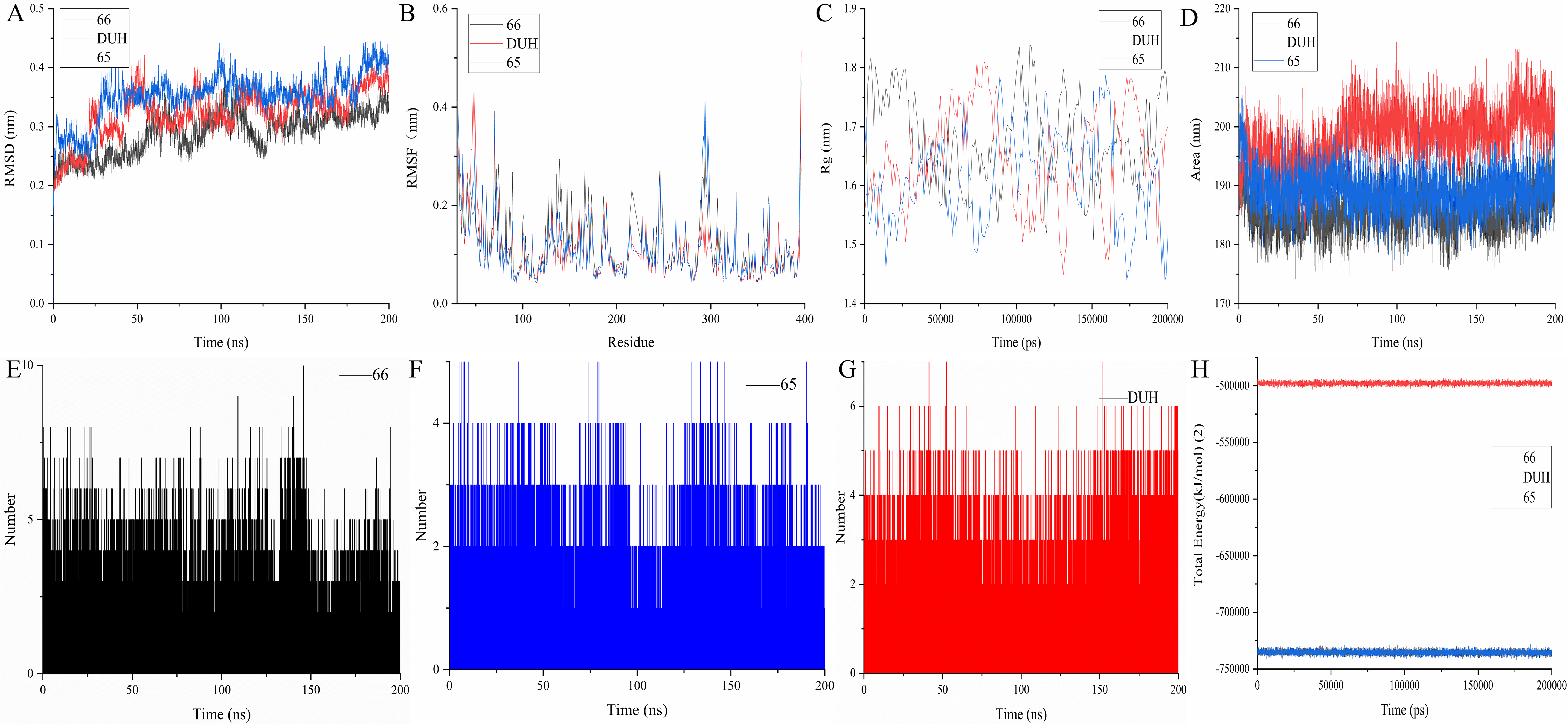

Supplement: S4 Fig — (A) The RMSD of compounds 65 and 66、DUH with DHODH. Compound 65 is shown as blue lines, compound 66 is in the black line. DUH is in the red line. (B) The RMSF of compounds 65 and 66 and DUH with DHODH. Compound 65 is shown as blue lines, compound 66 is in the black line. DUH is in the red line. (C) The Rg of compounds 65 and 66 and DUH with DHODH. Compound 65 is shown as blue lines, compound 66 is in the black line. DUH is in the red line. (D) The Area of compounds 65 and 66 and DUH with DHODH. Compound 65 is shown as blue lines, compound 66 is in the black line. DUH is in the red line. (E) The Hydrogen bond number of compound 65 with DHODH. (F) The Hydrogen bond number of compound 66 with DHODH. (G) The Hydrogen bond number of compound DUH with DHODH. (H) The total energy of compounds 65 and 66 and DUH with DHODH. Compound 65 is shown as blue lines, compound 66 is in the black line. DUH is in the red line. (TIF) [file pone.0342461.s006.tif]

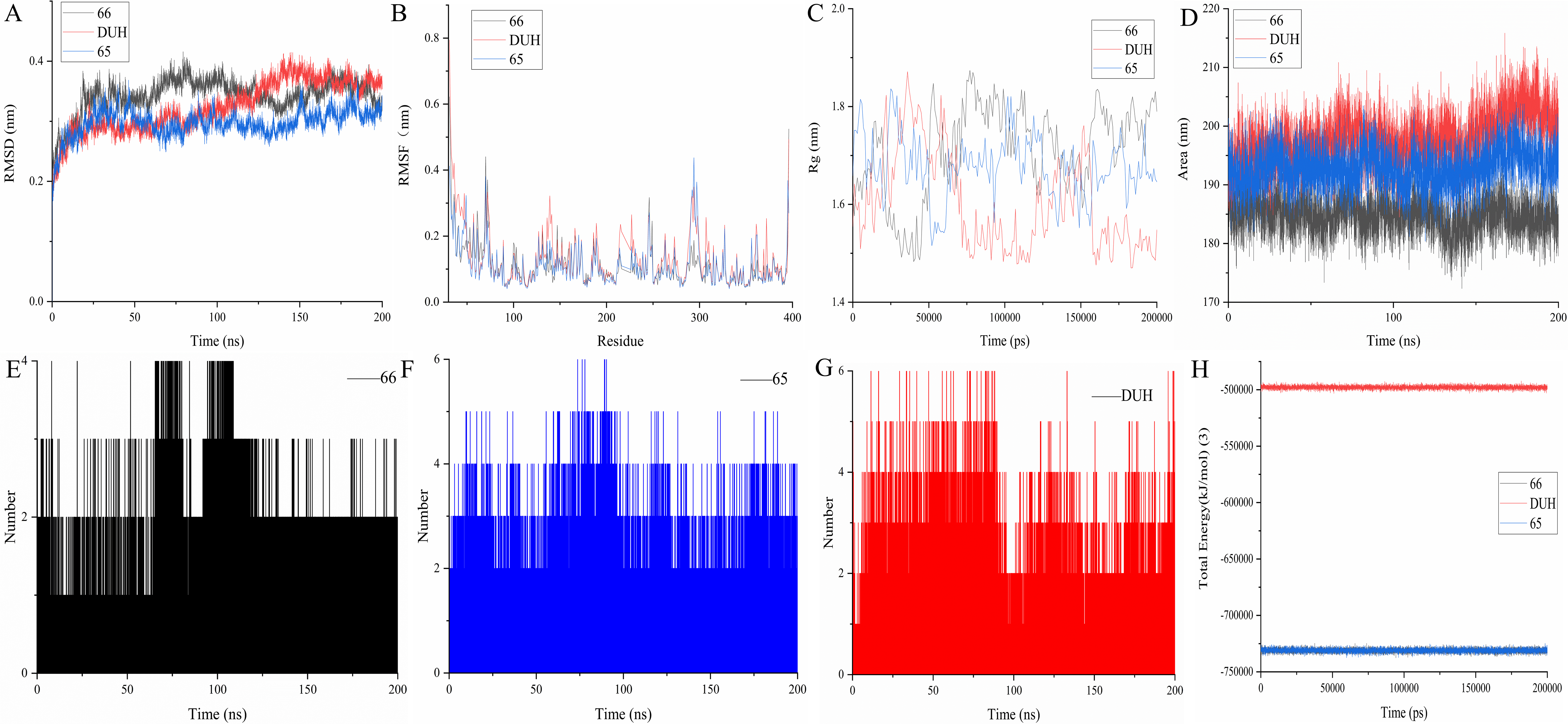

Supplement: S5 Fig — (A) The RMSD of compounds 65 and 66、DUH with DHODH. Compound 65 is shown as blue lines, compound 66 is in the black line. DUH is in the red line. (B) The RMSF of compounds 65 and 66 and DUH with DHODH. Compound 65 is shown as blue lines, compound 66 is in the black line. DUH is in the red line. (C) The Rg of compounds 65 and 66 and DUH with DHODH. Compound 65 is shown as blue lines, compound 66 is in the black line. DUH is in the red line. (D) The Area of compounds 65 and 66 and DUH with DHODH. Compound 65 is shown as blue lines, compound 66 is in the black line. DUH is in the red line. (E) The Hydrogen bond number of compound 65 with DHODH. (F) The Hydrogen bond number of compound 66 with DHODH. (G) The Hydrogen bond number of compound DUH with DHODH. (H) The total energy of compounds 65 and 66 and DUH with DHODH. Compound 65 is shown as blue lines, compound 66 is in the black line. DUH is in the red line. (TIF) [file pone.0342461.s007.tif]

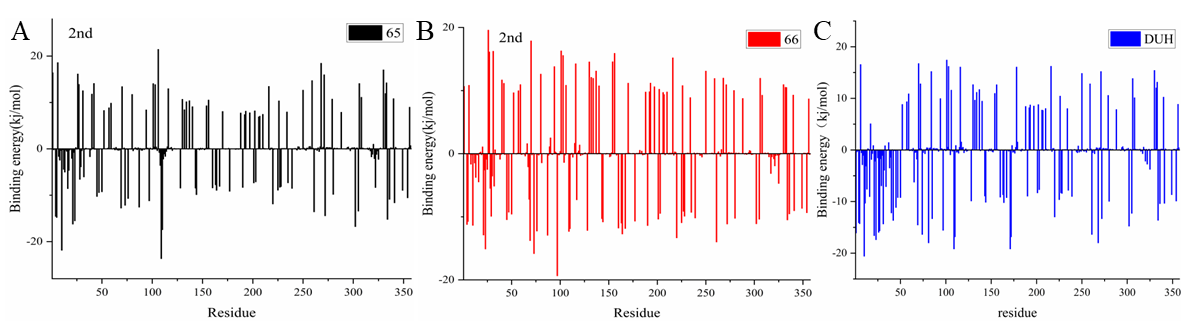

Supplement: S6 Fig — (A) Compound 65 with DHODH (black). (B) Compound 66 with DHODH (red). (C) Compound DUH with DHODH (blue). (TIF) [file pone.0342461.s008.TIF]

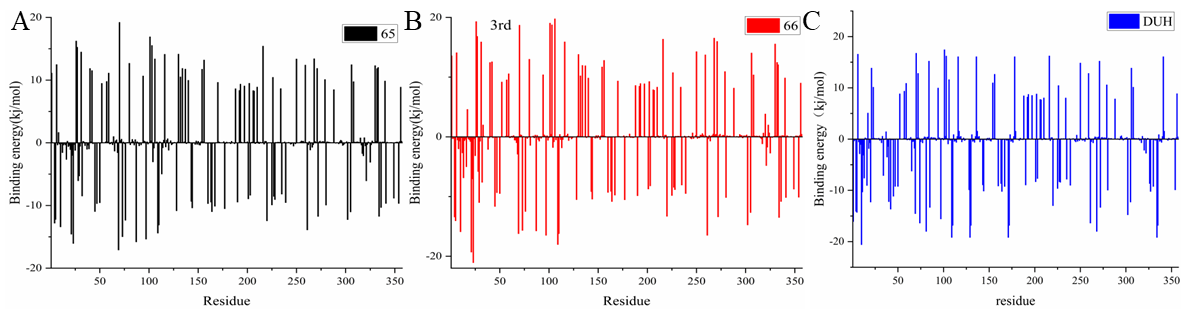

Supplement: S7 Fig — (A) Compound 65 with DHODH (black). (B) Compound 66 with DHODH (red). (C) Compound DUH with DHODH (blue). (TIF) [file pone.0342461.s009.TIF]

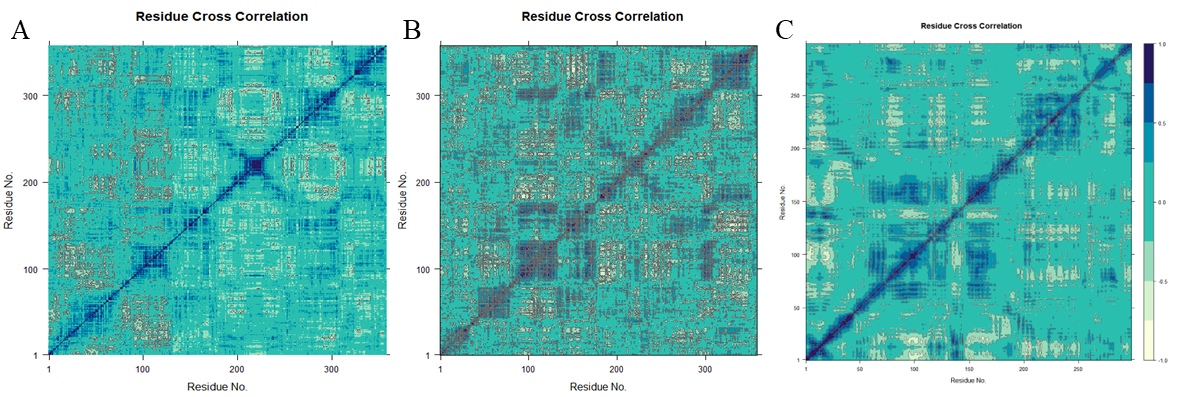

Supplement: S10 Fig — (A) Compound 65 with DHODH (B) Compound 66 with DHODH. (C) DUH with DHODH. Positive correlations between residues are shown in cyan and negative correlations are shown in light green. (TIF) [file pone.0342461.s012.TIF]

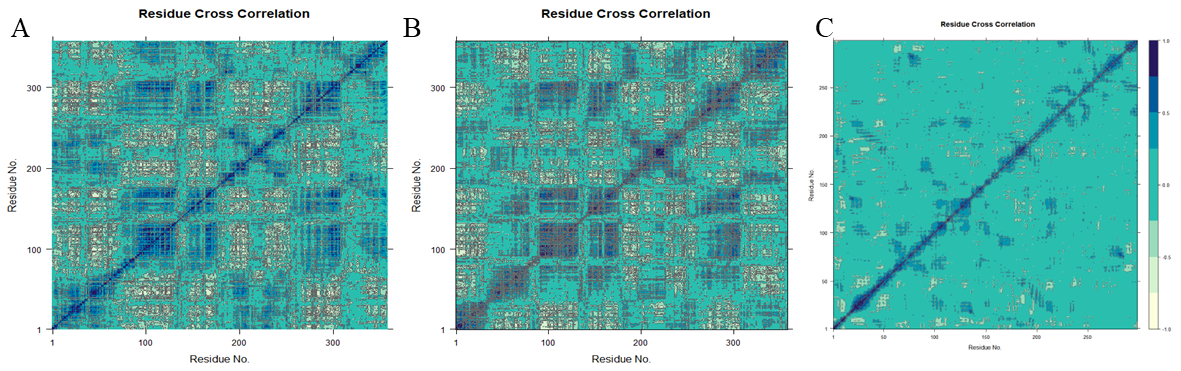

Supplement: S11 Fig — (A) Compound 65 with DHODH (B) Compound 66 with DHODH. (C) DUH with DHODH. Positive correlations between residues are shown in cyan and negative correlations are shown in light green. (TIF) [file pone.0342461.s013.TIF]

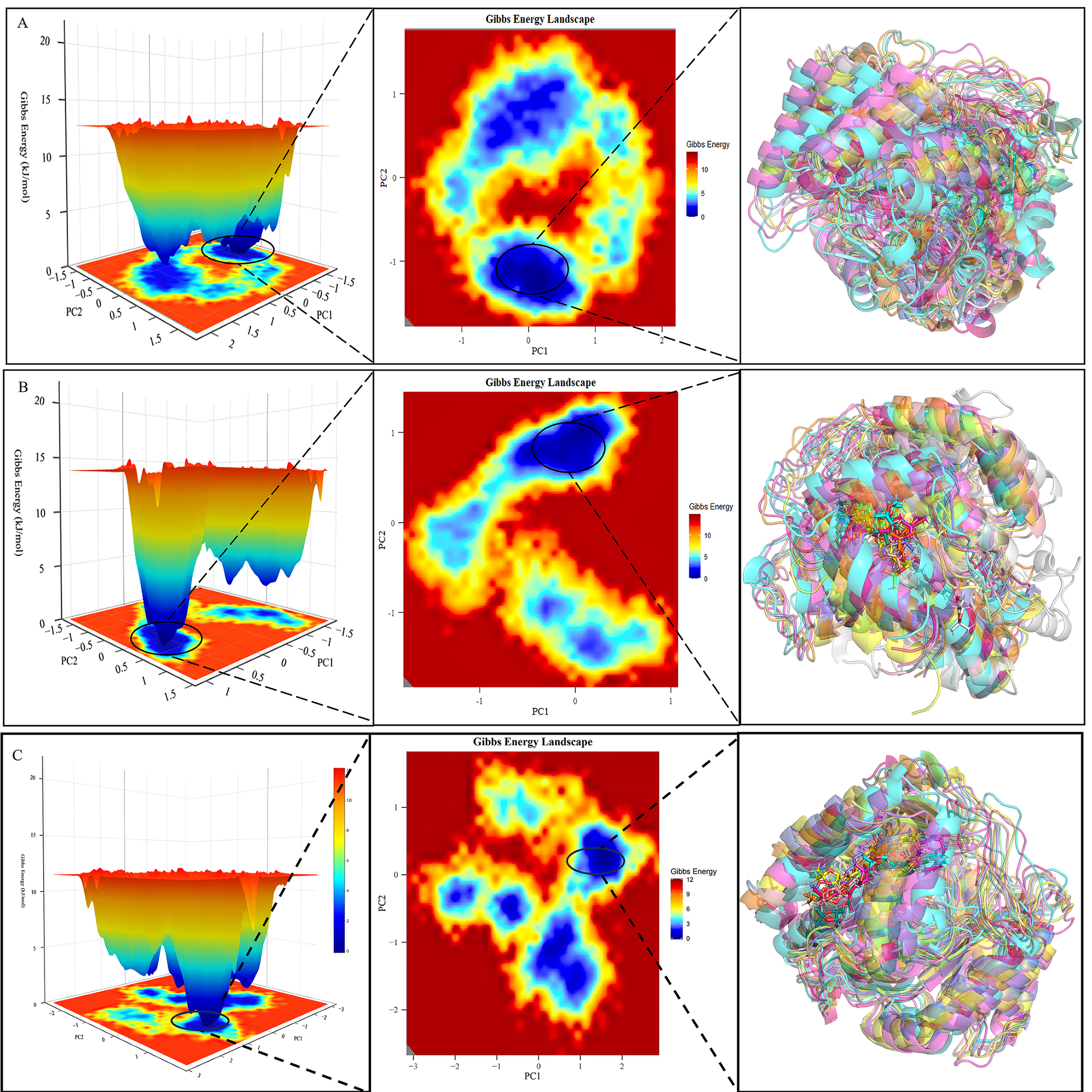

Supplement: S12 Fig — (A) Compound 65 with DHODH (B) Compound 66 with DHODH. (C) DUH with DHODH. (TIF) [file pone.0342461.s014.tif]

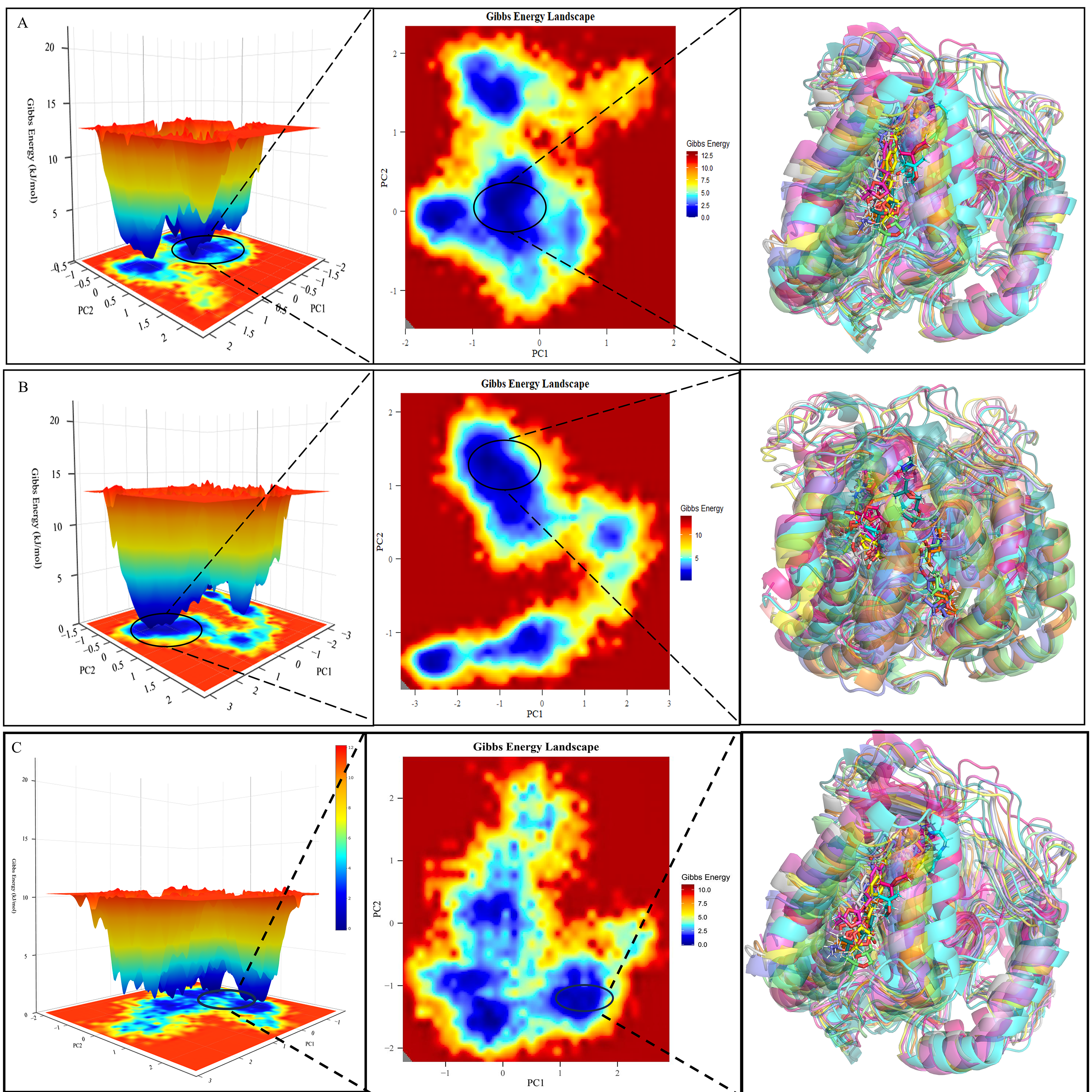

Supplement: S13 Fig — (A) Compound 65 with DHODH (B) Compound 66 with DHODH. (C) DUH with DHODH. (TIF) [file pone.0342461.s015.tif]
