# Supplementary material for: Development of DHODH inhibitors incorporating virtual screening, pharmacophore modeling, fragment-based optimization methods, ADMET, molecular docking, molecular dynamics, PCA analysis, and free energy landscape
Source: PLoS One. 2026 Feb 23;21(2):e0342461. doi: 10.1371/journal.pone.0342461 (PMC12928574; doi:10.1371/journal.pone.0342461)
Supplement: Attachment — Submitted filename: report-PONE-D-25-31041.pdf [file pone.0342461.s016.pdf]

Dear Editor,

I commend the authors for submitting the manuscript ***"Development of DHODH inhibitors incorporating virtual screening, pharmacophore modeling, fragment-based optimization methods, ADMET, molecular docking, molecular dynamics, PCA analysis, and free energy landscape"*** (ID PONE-D-25-31041). This study presents a computational perspective on drug design of DHODH inhibitors.

I have identified several significant weaknesses in the manuscript that must be addressed before it can be considered for publication in *PLOS ONE*. The manuscript is unsuitable for publication in its current form.

Major Points

1) **On Abstract:** Provide more quantitative results and analysis. No crucial result is presented.
2) **On Introduction:** According to the authors *"Numerous dihydroorotate dehydrogenase inhibitors (DHODH) have advanced to clinical trials to treat various oncological conditions"*; however, this claim is not supported by any references. Appropriate citations should be provided to substantiate this statement. The introduction should present the primary goal, as well as the tools used to achieve it.
3) **On Materials and Methods: 3.1 Most critical point:** The selected PDB structure (6FMD) contains FMN as a cofactor. However, it is unclear how this cofactor was treated in the classical simulations, including both molecular docking and molecular dynamics. The authors should clarify whether FMN was retained, removed, or parameterized, and how its presence may have influenced the results. **3.2** Proper reference for Discovery Studio. I suggest other programs for ADMET (e.g. SwissADME). **3.3** The authors should be more rigorous in describing the methodological procedures adopted. Some essential pieces of information are missing, such as the coordinates of the centre of mass and the size of the search box used in the molecular docking simulations; It's not clear how were docking procedures validated, so I recommend use a consensual docking procedure; How were pKa values computed for computational simulations (e.g. PROPKA method)? **3.3** To run MD of each system in triplicate (at least 200 ns each). The authors should explain how the selected ensemble from molecular dynamics (MD) simulations was chosen for binding free energy (BEF) analysis and whether it is statistically robust. **3.4** I recommend

including key analyses such as molecular fingerprints, followed by residual decomposition analysis; **3.5** Include DUH (crystal inhibitor) as a reference in all simulations.

Minor Points

1) To include PDB and input files as supplementary material.
2) English review
