# Supplementary material for: Development of DHODH inhibitors incorporating virtual screening, pharmacophore modeling, fragment-based optimization methods, ADMET, molecular docking, molecular dynamics, PCA analysis, and free energy landscape
Source: PLoS One. 2026 Feb 23;21(2):e0342461. doi: 10.1371/journal.pone.0342461 (PMC12928574; doi:10.1371/journal.pone.0342461)
Supplement: Attachment — Submitted filename: report-PONE-D-25-31041-R1.pdf [file pone.0342461.s018.pdf]

Dear Editor,

In the revised version of the manuscript entitled **“Development of DHODH inhibitors incorporating virtual screening, pharmacophore modeling, fragment-based optimization methods, ADMET, molecular docking, molecular dynamics, PCA analysis, and free energy landscape”** (ID: PONE-D-25-31041R1), the authors have only partially addressed my previous concerns. Unfortunately, the most critical issue remains unresolved. I have also identified several additional weaknesses that must be carefully addressed before the manuscript can be considered for publication in PLOS ONE. In its current form, the manuscript remains unsuitable for publication.

Major Points

1) **On Materials and Methods: 1.1 Most critical point:** The selected PDB structure (6FMD) contains FMN as a cofactor. However, it is unclear how this cofactor was treated in the classical simulations, including both molecular docking and molecular dynamics. The authors should clarify whether FMN was retained, removed, or parameterized, and how its presence may have influenced the results. **Author's Reply:** *Thank you for the constructive comments. For the FMN structure and others, we had already cleaned and removed water from the 6FMD structure on PyMOL before the docking. Therefore, we do not need the FMN structure.* **However, FMN is an essential cofactor and cannot be omitted from the computational model. Its exclusion renders the systems unrealistic. No references for PDB code and docking softwares applied. No units for XYZ coordinates.** **1.2** Include DUH (crystal inhibitor) as a reference in all simulations. **Not attended by authors.**
